# A rare case of low-grade myofibroblastic sarcoma of the femur in a 38-year-old woman: a case report

**DOI:** 10.1186/1752-1947-4-121

**Published:** 2010-04-28

**Authors:** Raman Arora, Ruchika Gupta, Alok Sharma, Amit K Dinda

**Affiliations:** 1 Department of Pathology, All India Institute of Medical Sciences, Ansari Nagar, New Delhi, India

## Abstract

**Introduction:**

Primary myofibroblastic sarcoma of the bone is a rare spindle cell tumour with, to the best of our knowledge, only eight cases reported in the available English language literature. The disease's rarity and its low-grade features make an accurate diagnosis difficult in most cases. The differential diagnoses of this unusual tumour include various benign entities as well as other sarcomas. Due to the difference in prognosis, a precise pathologic diagnosis is essential, which requires a combination of thorough morphologic examination, immunohistochemistry and electron microscopy wherever available.

**Case presentation:**

We report the case of a 38-year-old Indian woman with a lytic lesion in her left femur. The tumour was associated with cortical destruction and soft tissue extension. A biopsy from the soft tissue component showed features suggestive of a low-grade malignant mesenchymal tumour. Excision of the tumour was performed and histopathological examination showed a low-grade spindle cell sarcoma with collagenous stroma. Expressions of vimentin and smooth muscle actin were also noted. Ultrastructural examination confirmed its myofibroblastic nature. A final diagnosis of low-grade myofibroblastic sarcoma of the left femur was thus rendered.

**Conclusion:**

Low-grade myofibroblastic sarcoma is one of the rarer osseous spindle cell sarcomas depicting a favourable prognosis in the cases reported so far. Its diagnosis requires ancillary techniques like immunohistochemistry and electron microscopy. To the best of our knowledge, we report the ninth case in the literature and the first case from our subcontinent.

## Introduction

Myofibroblasts are mesenchymal cells showing characteristics of both fibroblasts and smooth muscle cells. In addition to its role in wound healing, they have been described in soft tissue tumours like myofibroblastoma, angiomyofibroblastoma, myofibromatosis and inflammatory myofibroblastic tumour [1,2]. Myofibroblastic sarcoma was characterized as a distinct neoplasm in 1998 by Mentzel *et al*. [3]. Primary myofibroblastic sarcoma of the bone is rare with only eight cases reported in the available English language literature [3-8]. To the best of our knowledge, no such case has been reported from our subcontinent.

The histopathological differential diagnoses of this rare neoplasm include benign myofibroblastic proliferations and sarcomas such as well-differentiated intraosseous osteosarcoma, leiomyosarcoma, fibrosarcoma and malignant fibrous histiocytoma of the bone [9-11]. An accurate diagnosis is essential since low-grade myofibroblastic sarcoma has a favourable prognosis compared to other osseous sarcomas. This requires the use of ancillary techniques like immunohistochemistry.

We describe a case of low-grade myofibroblastic sarcoma occurring in the femur of a 38-year-old woman. This rare entity is briefly reviewed with a discussion of various differential diagnoses.

## Case presentation

A 38-year-old Indian woman presented in our hospital with a two-year history of swelling and pain in her left thigh and hip. The swelling was progressive in nature. She had no history of trauma prior to the swelling. No significant personal or family history was present. On examination, there was a 20 × 10 cm soft tissue swelling involving the anterolateral aspect of her left thigh and extending to her hip. There was mild tenderness over the swelling and movements at her hip joint were painfully restricted. Results of systemic examination, however, were unremarkable.

**Figure 1:**
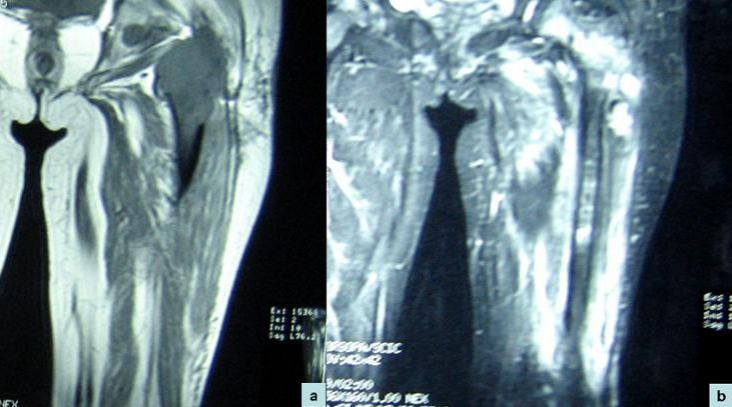
**Magnetic resonance imaging shows evidence of lobulated soft tissue mass involving the upper end of left femur**. **(A) **The mass was hypointense on T1-weighted imaging and **(B) **hyperintense on short inversion recovery imaging.

Routine haematological and biochemical investigations, including alkaline phosphatase, were within reference ranges. Radiological investigations, such as computed tomography (CT) scan and magnetic resonance imaging (MRI), revealed a diffuse, irregular, ill-defined heterogeneous altered marrow signals in our patient's left upper femoral metaphysis and extending into the epiphysis and diaphysis. The altered marrow signals were seen to extend up to the lower shaft diaphysis and metaphysis. Similar signals were also noted in her left pelvic bone (acetabulum and pubic). There was anterior and posterior cortical disruption in her upper femoral metaphysic with extension into the adjacent soft tissues. The altered marrow signals were hyperintense on T2-weighted and short inversion recovery (STIR) images. On the other hand, the signals were low on T1-weighted images (Figure 1).

A bone scan confirmed these findings. No other skeletal lesions were likewise detected. The radiological features were suggestive of a malignant neoplasm. A core biopsy from the soft tissue swelling, which was performed at another hospital, was reviewed at our institute and showed a spindle cell tumour with focal nuclear pleomorphism, intercellular collagenous stroma, and occasional mitotic figures. With an initial diagnosis of malignant mesenchymal tumour, our patient underwent proximal femoral resection and prosthesis reconstruction.

**Figure 2:**
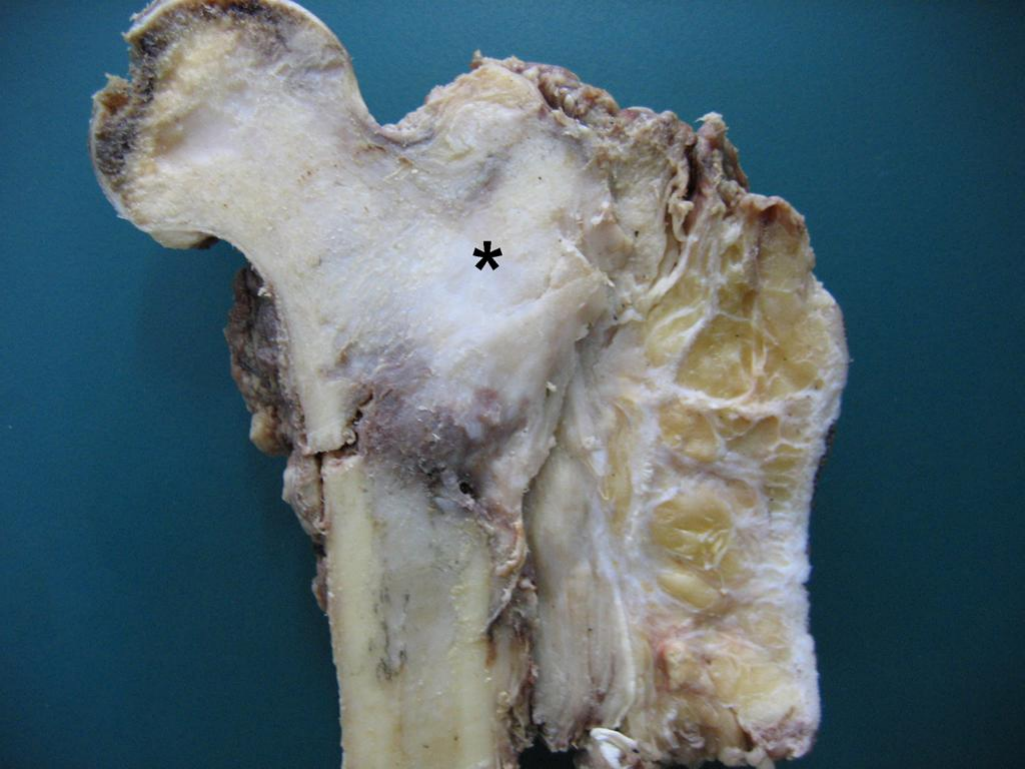
**Gross specimen of the proximal femur showing a grey-white firm tumuor (asterisk) with destruction of the adjacent cortex**.

**Figure 3:**
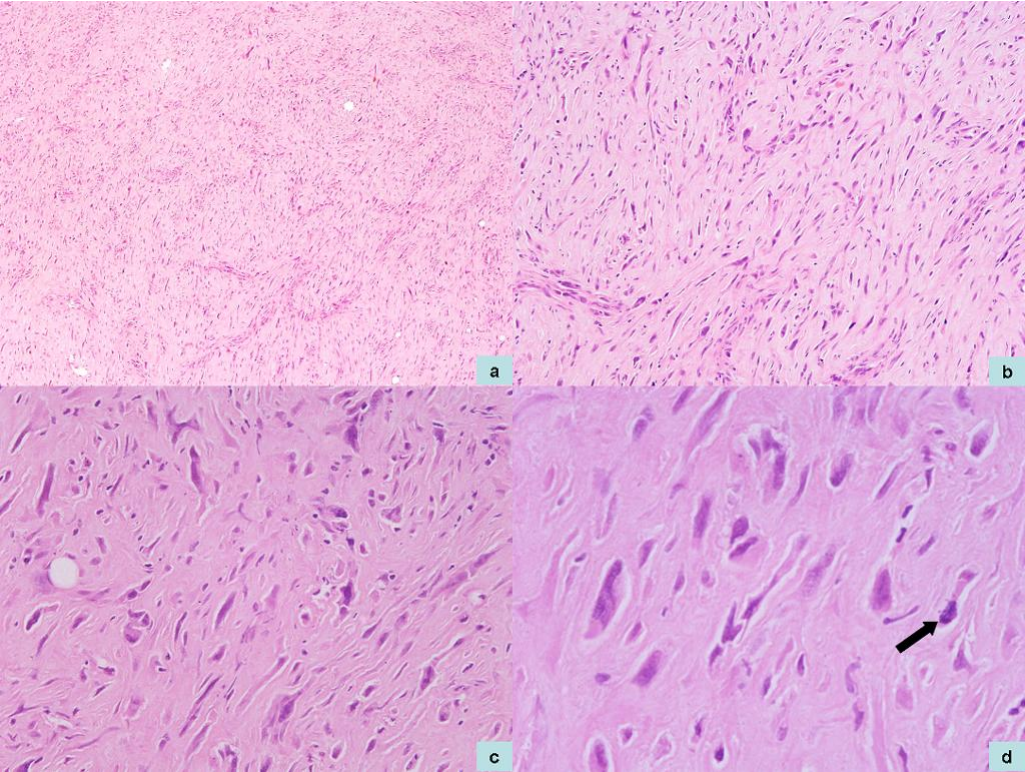
**(A) Photomicrographs demonstrating a spindle cell tumour with interspersed hypocellular areas (Hematoxylin and Eosin stain, ×40 magnificaiton)**. **(B) **The spindle cells show moderate degree of nuclear pleomorphism (Hematoxylin and Eosin stain, ×100 magnification). **(C) **Occasional bizarre tumour cells (Hematoxylin and Eosin stain, ×200 magnificaiton). **(D) **An occasional mitotic figure was noted (Hematoxylin and Eosin stain, ×400 magnificaiton).

We received our patient's proximal femur measuring 13 × 9 × 7 cm with a fusiform swelling, with 8 × 7 × 4 cm of the femur involving metaphysis. The tumour was seen to destroy the cortex and was extending into the soft tissue (Figure 2). Multiple sections from the tumour showed a spindle cell lesion composed of hypercellular fascicular and hypocellular fibrous areas (Figure 3A). The hypercellular foci showed spindle cells arranged in short fascicles and focally in a storiform pattern. The spindle cells had abundant eosinophilic cytoplasm and elongated nuclei. It also had finely dispersed chromatin and inconspicuous nucleoli. There was moderate nuclear pleomorphism and some nuclei showed indented nuclear membranes (Figure 3B and 3C). Occasional bizarre nuclei and a mitotic count of 1 to 2 per 10 high power fields (HPF) were also seen (Figure 3D). There was no osteoid deposition, necrosis, or multinucleated giant cells in the numerous sections studied.

**Figure 4:**
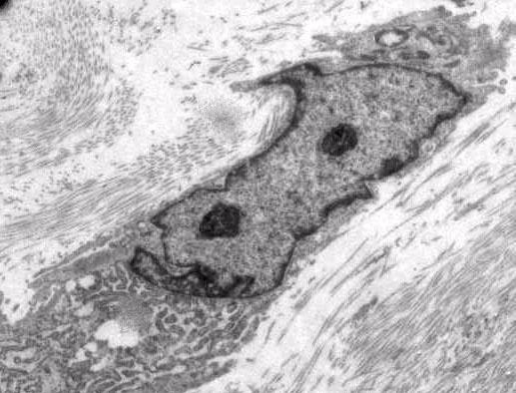
**Electron photomicrograph showing a tumour cell with prominent endoplasmic reticulum and surrounded by collagen fibrils (Uranyl acetate and Lead citrate ×13,000)**.

The spindle cells were found to be positive for vimentin and smooth muscle actin and negative for desmin, S-100 protein, myogenin and CD68 on immunohistochemistry. MIB-1 labeling index was low at 1% to 2%. Ultrastructural examination showed spindle-shaped cells with abundant rough endoplasmic reticulum, scattered mitochondria, and longitudinally arranged microfilaments. This was suggestive of a myofibroblastic nature (Figure 4).

The histomorphological, immunohistochemical and ultrastructural features suggested a final diagnosis of low-grade myofibroblastic sarcoma of the left upper femur. Our patient is currently on follow-up with no appreciable increase in the residual tumour.

## Discussion

Myofibroblasts have been characterized in the last three decades, as mesenchymal spindle cells sharing features of both fibroblasts and smooth muscle cells. These cells have been shown to play an important role in wound healing [4]. In addition, myofibroblasts have been recognized in pseudosarcomatous proliferations, hypertrophic scars, and superficial and deep fibromatoses [3]. Over the last few years, neoplasms including infantile digital fibromatosis, myofibromatosis, mammary myofibroblastoma, dermatomyofibroma, cutaneous myofibroma and angiomyofibroblastoma have been described as composed primarily of myofibroblasts [1]. In 1998, Mentzel *et al*. characterized myofibrosarcoma or myofibroblastic sarcoma as a spindle cell sarcoma composed of myofibroblasts. In their study, they showed that the myofibroblasts stained positively for at least one of the myogenic markers (desmin, smooth muscle actin, and smooth muscle myosin heavy chain) and vimentin [3].

Histopathologically, myofibroblastic sarcomas (MFS) are composed of slender spindle cells with variable nuclear pleomorphism and mitotic activity. The spindle cells are arranged in interlacing fascicles and have eosinophilic cytoplasm, which may be occasionally wavy. In addition, collagenous stroma is also seen [2,6,12]. Low-grade MFS (LGMFS) lacks necrosis and prominent nuclear pleomorphism, as seen in our patient [3].

Primary MFS of the bone is an extremely rare neoplasm. An extensive literature search yielded only eight previously reported cases of osseous MFS [3-8]. Of the reported cases, the most common location was the femur, followed by the iliac bone. The tumours have occurred over a wide age range with no gender predilection. Meanwhile, therapeutic approaches in these cases have varied from simple curettage to amputation with some patients receiving radiotherapy. Of the eight patients reported, six have done well without local recurrence or distant metastasis in follow-up periods ranging from 2 to 16 years.

Two patients (a 71-year-old woman with iliac bone tumour and a 49-year-old man with tumour in the right maxilla) died with distant metastasis. These two patients showed a high mitotic count (6 to 8 per 10 HPF) and necrosis. However, these histopathological features did not correlate well with prognosis, since some cases with high mitotic activity and marked atypia pursued an indolent course. Both patients who died of their disease were treated by wide resection with radiotherapy and/or chemotherapy. Due to the unpredictable behaviour of their disease, these patients had to be kept under close follow-up, especially because the histopathological features of the disease do not seem to predict accurately its biologic behaviour. The reported cases of osseous MFS are summarised in Table 1.

**Table 1:** Summary of reported cases of myofibroblastic sarcoma of the bone.

Authors	No. of cases	Age/Gender	Site of tumour	Size of tumour	Therapy	Follow-up
Majno G[5]	1	49/M	Jaw	8 cm	WR + RT	DOD

Bisceglia *et al*.[2]	1	24/M	Jaw	4 cm	WR	AWOD

Montgomery *et al*.[8]	1	65/M	Tibia	6 cm	WR + Amp	AWOD

Watanabe *et al*.[11]	4	60/M	Femur	5 cm	WR + CT	AWOD
		63/W	Femur	9 cm	Curettage	AWOD
		66/W	Ilium	9.5 cm	WR + CT	AWOD
		71/W	Ilium	7 cm	WR + CT	DOD

San Miguel *et al*.[9]	1	51/W	Distal phalanx	1.8 cm	Local excision	AWOD

Present case	1	38/W	Femur	8 cm	Resection	AED

M, man; W, woman; WR, wide resection; CT, chemotherapy; Amp, amputation; RT, radiotherapy; DOD, dead of disease; AWOD, alive without disease; AED, alive with evidence of disease.

MFS needs to be differentiated from tumours like conventional osteosarcomas, chondroblastoma and solitary fibrous tumour, which may show focal or extensive myoid differentiation. These tumours can be differentiated by their clinicopathological features [11]. Another tumour, well-differentiated intraosseous osteosarcoma, may be confused with MFS since the former may be composed predominantly of fibrous tissues [9]. Well-differentiated osteosarcoma occurs in adolescents and young adults as a bone-forming tumour (evident on radiology and histology) with less cellular atypia than MFS.

Other neoplasms that enter the list of differential diagnoses include leiomyosarcoma, fibrosarcoma, and malignant fibrous histiocytoma of the bone. Leiomyosarcoma, whether primary or metastatic, can be differentiated by its lack of prominent collagenous stroma and expression of h-caldesmon [11]. Fibrosarcoma of the bone has morphological similarities to LGMFS. These include wavy spindled cytoplasm, collagenous stroma, and wide spectrum of cellular anaplasia. However, LGMFS can be distinguished by the immunohistochemical expression of myoid antigens [2]. Unlike LGMFS, on the other hand, malignant fibrous histiocytoma (MFH) shows marked cellular anaplasia with multinucleated giant cells [10].

## Conclusion

Low-grade myofibroblastic sarcoma is an unusual osseous neoplasm. It needs to be differentiated from other spindle cell tumours of the bone using immunohistochemical expression pattern and/or electron microscopy. A close radiological and pathological correlation is mandatory to exclude other entities and to make an accurate diagnosis. The biologic behaviour of this rare neoplasm is difficult to predict because only a few cases have been reported so far. Reports of more cases in the literature would help clinicians define this entity better.

## Consent

Written informed consent was obtained from our patient for publication of this case report and any accompanying images. A copy of the written consent is available for review by the Editor-in-Chief of this journal.

## Competing interests

The authors declare that they have no competing interests.

## Authors' contributions

RA reported the histopathological specimen and collected the clinical data. RG reviewed the literature and drafted the manuscript. AS assisted in drafting and revising the manuscript. AKD was in-charge of signing out the case and critically revised the manuscript. All authors read and approved the final manuscript.
